# Empathic Listening and Communication Competencies Among Oncology Healthcare Professionals in Croatia: A Cross-Sectional Study Conducted in 2025

**DOI:** 10.3390/healthcare14131842

**Published:** 2026-06-24

**Authors:** Sandra Karabatić, Marin Mamić, Božica Lovrić, Vajdana Tomić, Stjepan Orešković

**Affiliations:** 1 University Hospital Centre Zagreb, Kišpatićeva 12, 10000 Zagreb, Croatia; 2 Faculty of Health Studies, University of Mostar, 88000 Mostar, Bosnia and Herzegovina; vajdana.tomic@fzs.sum.ba; 3 Department of Nursing, Catholic University of Croatia, Ilica 242, 10000 Zagreb, Croatia; 4 Department of Nursing, University of Applied Health Sciences, Mlinarska Cesta 38, 10000 Zagreb, Croatia; 5 JEDRA Association, Jordanovac 104, 10000 Zagreb, Croatia; 6 General County Hospital Požega, Osječka 107, 34000 Požega, Croatia; bozica.lovric@pozeska-bolnica.hr; 7 Faculty of Dental Medicine and Health Osijek, Josip Juraj Strossmayer University of Osijek, Crkvena 21, 31000 Osijek, Croatia; 8 Department of Nursing, University of Applied Sciences Ivanić-Grad, Moslavačka 13, 10310 Ivanić-Grad, Croatia; 9 School of Medicine, University of Zagreb, Šalata 3, 10000 Zagreb, Croatia; soreskov@snz.hr

**Keywords:** patient-centered communication, oncology, communication, empathy, healthcare professionals, professional competence, communication skills, quality of healthcare

## Abstract

**Introduction/Objectives:** Patient-centered communication is essential in oncology care, where healthcare professionals often manage emotionally demanding conversations, uncertainty, complex decisions, and patient involvement in care. However, the relationship between communication knowledge, empathic listening, and practical communication skills remains insufficiently examined. This study aimed to examine the associations between communication knowledge, empathic listening, and interpersonal communication skills among healthcare professionals involved in oncology care. **Methods:** A cross-sectional study was conducted in Croatia from May to November 2025 on a convenience sample of 138 healthcare professionals involved in oncology care. Communication knowledge was assessed using a study-specific questionnaire, empathic listening using an adapted Active Empathic Listening Scale, and interpersonal communication skills using an adapted Interpersonal Communication Skills Inventory. Because the instruments were adapted to the oncology care context, their dimensions were examined using exploratory factor analysis and interpreted as sample-specific exploratory constructs. Descriptive statistics, correlation analyses, and multiple linear regression analyses were performed. **Results:** Clear message delivery and assertiveness had the highest self-reported score, whereas emotional interaction management had the lowest. Communication knowledge was not an independent predictor of communication skills dimensions. Processing and responding positively predicted clear message delivery and assertiveness (β = 0.361; *p* = 0.001; R^2^ = 13.4%), while noticing emotional and nonverbal cues negatively predicted emotional interaction management (β = −0.234; *p* = 0.032; R^2^ = 7.6%). The explained variance of the models was low. **Conclusions:** The findings suggest limited but potentially relevant associations between selected dimensions of empathic listening and self-reported communication skills in oncology care. Communication knowledge, measured using a study-specific exploratory instrument, was not independently associated with communication skills. Because of the exploratory design, self-report measures, adapted instruments, and convenience sampling, the results should be interpreted with caution.

## 1. Introduction

Patient-centered communication is an important component of high-quality healthcare because it contributes to patients’ understanding of health information, trust, satisfaction with care, adherence to treatment, and involvement in decision-making [1,2]. In contemporary healthcare, communication is not viewed only as the transfer of information but as an interactive process through which healthcare professionals establish relationships with patients, respond to their needs, and support shared understanding of health problems [3,4]. Communication is also linked to broader healthcare outcomes, including patient experience, decision-making, and the quality of the patient–healthcare professional relationship [5].

Communication is particularly important in oncology, where healthcare professionals and patients often face emotionally demanding conversations, uncertainty about treatment outcomes, complex therapeutic decisions, and issues related to prognosis, treatment side effects, and end-of-life care [6,7]. In such situations, communication requires not only the clear exchange of medical information but also recognition of emotional reactions, empathic responses, shared decision-making, and support for patient participation in care [8]. Therefore, communication competencies are an important factor in the quality and safety of patient-centered oncology care.

Communication competence is a multidimensional construct that includes communication knowledge, interpersonal communication skills, emotional sensitivity, reflection, and the ability to adapt communication to the needs of an individual patient [9,10]. Communication knowledge refers to understanding communication principles, processes, and strategies, whereas interpersonal communication skills reflect the ability to apply this knowledge in real clinical situations. This distinction is important because declarative knowledge does not necessarily result in effective communication in emotionally complex situations such as those commonly encountered in oncology care [11].

Empathic listening is an important relational component of communication competence. It includes the ability to recognize verbal, nonverbal, and emotional messages, process the information conveyed by the patient, and respond appropriately [12]. In oncology care, empathic listening may help healthcare professionals recognize patients’ concerns, understand emotional reactions to illness, and adapt communication to individual patient needs. Previous research has shown that empathy and empathic communication are associated with higher patient satisfaction, lower psychological distress, and better healthcare experiences [13,14]. Related concepts, such as compassion and emotionally supportive communication, are also important when patients are faced with vulnerability, fear, and uncertainty [15].

Oncology communication is especially challenging in situations such as breaking bad news, discussing prognosis, and supporting patients during treatment [16,17]. Structured approaches, such as the SPIKES protocol, emphasize preparation, assessment of patients’ understanding, information sharing, responses to emotions, and agreement on next steps [18]. These approaches show that communication competence depends not only on theoretical knowledge but also on the ability to apply communication principles in emotionally demanding clinical situations.

Communication skills education is therefore recommended for healthcare professionals working with oncology patients. Previous research indicates that communication competence can be improved through simulations, structured feedback, reflective practice, and experiential learning, rather than through the transfer of theoretical knowledge alone [19,20]. Training programs are more effective when they include active teaching methods and repeated practice, while passive forms of training often have a limited impact on actual professional behavior [21,22]. Reflective practice may additionally help healthcare professionals connect knowledge, experience, and the emotional demands of clinical communication [23,24].

However, it remains insufficiently clear how communication knowledge, empathic listening, and self-assessed interpersonal communication skills are interrelated among healthcare professionals involved in oncology care. In particular, previous research has not sufficiently clarified whether practical communication skills are more strongly associated with declarative communication knowledge or with relational competencies such as empathic listening. This gap is relevant because oncology communication training programs usually include both theoretical and experiential components, yet it remains unclear which components are more closely related to self-assessed practical communication skills. At the same time, such relationships should be interpreted with caution, especially when communication competencies are assessed using self-report instruments and instruments adapted to specific clinical and cultural contexts.

Therefore, the aim of this study was to examine the association between communication knowledge, empathic listening, and interpersonal communication skills among healthcare professionals involved in oncology care in Croatia. The specific objectives were to examine the association between dimensions of communication knowledge and empathic listening with dimensions of interpersonal communication skills and to determine the extent to which communication knowledge and empathic listening explain individual dimensions of self-assessed interpersonal communication skills. The hypothesis was that empathic listening would be positively associated with interpersonal communication skills, whereas communication knowledge would show weaker and less consistent associations with practical communication skills.

## 2. Materials and Methods

### 2.1. Study Design

A cross-sectional study was conducted among healthcare professionals involved in oncology care in the Republic of Croatia from May to November 2025. Data were collected using an anonymous online questionnaire. The study was conducted in accordance with the STROBE guidelines for reporting cross-sectional studies [25].

### 2.2. Participants and Data Collection Procedure

Convenience sampling was used. Healthcare professionals who participated in diagnostic, therapeutic, educational, advisory, or other forms of care for oncology patients were eligible to participate. The inclusion criteria were healthcare professional status, experience in oncology care, and voluntary consent to participate. The exclusion criteria were non-involvement in oncology care, failure to meet the eligibility criteria, and incomplete data for the main variables included in the analyses.

The link to the questionnaire was distributed electronically through the official communication channels of the Croatian Society of Oncology Nurses and Technicians, the Croatian Society of Internal Oncologists, and the Croatian Oncology Society. A total of 350 invitations to participate were sent. A total of 138 fully completed questionnaires were received, corresponding to a response rate of 39.4%.

Before accessing the questionnaire, participants were informed about the purpose of the study, voluntary participation, anonymity, data protection, and the possibility of withdrawing before submitting their answers. Informed consent was provided electronically, and access to the questionnaire was possible only after consent had been confirmed. The questionnaire was created in Google Forms with the option to limit responses to one per user in order to reduce the possibility of multiple submissions. Data were collected on age, gender, profession, education level, total years of work experience, and length of work in oncology care.

The required sample size was estimated using G*Power 3.1 for multiple linear regression with four predictors [26]. Assuming a medium effect size (f^2^ = 0.15), a statistical significance level of α = 0.05, and a power of 80%, the minimum required sample size was 85 participants. Since 138 participants were included in the study, including 136 participants with complete data for regression analyses, the sample size was considered sufficient for the planned exploratory regression analyses.

### 2.3. Research Instruments and Adaptation Procedure

Three instruments were used to assess communication knowledge, empathic listening, and interpersonal communication skills. Before use in the main study, the instruments were linguistically and culturally adapted. The original items were translated into Croatian and then back-translated into the original language to check semantic equivalence. Minor linguistic differences between the original and back-translated versions were resolved by consensus among the authors.

The items were then contextually and terminologically adapted to oncology care so that they would be understandable to healthcare professionals working with oncology patients. The adapted versions were reviewed by an expert committee consisting of healthcare professionals with experience in oncology, communication skills, and research methodology. After expert review, a pilot study was conducted on a smaller sample of healthcare professionals to assess item comprehensibility, clarity of instructions, and questionnaire acceptability. Based on the feedback received, minor linguistic and content refinements were made before administration in the main study.

Since the instruments had not previously been validated among healthcare professionals involved in oncology care in Croatia, their structure was examined using exploratory factor analysis. The obtained dimensions were used as empirically derived indicators of the constructs included in the study and should be viewed as exploratory and sample-specific, not as final psychometric validation of the instruments. Detailed exploratory factor analysis results, including Kaiser–Meyer–Olkin measures, Bartlett’s tests of sphericity, eigenvalues, explained variance, pattern matrices, component correlation matrices, reliability coefficients, and excluded items, are provided in the Supplementary Materials.

#### 2.3.1. Communication Knowledge Questionnaire

Communication knowledge was assessed using a structured questionnaire developed for the purposes of this study. The items were designed based on literature related to patient-centered communication, communication in oncology care, breaking bad news, empathic communication, and communication skills education [2,8,16,17,18,19,20].

The questionnaire consisted of 20 dichotomous items. A correct answer was scored with one point, and an incorrect or “I do not know” answer with zero points. Due to the dichotomous nature of the items, exploratory factor analysis was conducted using a tetrachoric correlation matrix. The suitability of the data for factor analysis was assessed using the Kaiser–Meyer–Olkin measure and Bartlett’s test of sphericity. The number of factors was determined based on eigenvalues, scree plot inspection, theoretical interpretability, and item factor loadings.

After exploratory factor analysis, 17 items were retained in the final scoring of the Communication Knowledge Questionnaire. Three items were excluded because they did not meet the predefined factor loading criterion of ≥0.30 or did not contribute to a clear and interpretable factor structure. Two dimensions were identified: knowledge of communication theory and principles and knowledge of communication processes and skills. The complete pattern matrix and the list of excluded items are provided in the Supplementary Materials.

Reliability was assessed using McDonald’s omega coefficient. The knowledge of communication theory and principles dimension contained seven items and showed excellent internal consistency (ω = 0.981), while the knowledge of communication processes and skills dimension contained ten items and also showed high internal consistency (ω = 0.876). The total 17-item Communication Knowledge Questionnaire showed high reliability (ω = 0.909). Since the instrument was developed for the purposes of this study and has no previous external validation, the results obtained with this instrument should be interpreted as exploratory indicators of declarative communication knowledge in this sample.

#### 2.3.2. Interpersonal Communication Skills Inventory

Interpersonal communication skills were assessed using an adapted version of the Interpersonal Communication Skills Inventory by Armstrong [26]. The original version of the instrument contained 40 items. After linguistic and content adaptation, the instrument was applied in the context of oncology care, and its factor structure was examined using exploratory factor analysis.

Principal axis factoring with oblique oblimin rotation was used because the analysis was exploratory and because the extracted communication skill dimensions were expected to be conceptually related. Although the items were ordinal, the analysis was interpreted cautiously and the obtained structure was considered exploratory and sample-specific. The suitability of the data for factor analysis was assessed using the Kaiser–Meyer–Olkin measure and Bartlett’s test of sphericity. The KMO value was 0.596 for the initial 40-item version and 0.615 for the final 33-item version, indicating acceptable but modest sampling adequacy. Bartlett’s test of sphericity was statistically significant, supporting the factorability of the correlation matrix.

The initial application of Kaiser’s criterion, based on eigenvalues greater than 1, suggested an overly fragmented solution with several factors containing a small number of items. Therefore, the final number of factors was determined by combining eigenvalues, scree plot inspection, factor interpretability, and theoretical coherence. Items with acceptable factor loadings were retained, while items with low loadings, unclear loadings, or content that was not interpretable within the factor structure were omitted from further analysis.

After exploratory factor analysis, 33 items distributed across four dimensions were retained: clear message delivery and assertiveness, active listening, communication flow and feedback management, and emotional interaction management. The full pattern matrix, including retained and excluded items, is presented in the Supplementary Materials to provide transparency regarding factor loadings and item allocation.

The reliability of the subscales was assessed using McDonald’s omega coefficient. Omega coefficient values were 0.720 for clear message delivery and assertiveness, 0.611 for active listening, 0.604 for communication flow and feedback management, and 0.653 for emotional interaction management. The total reliability of the instrument was ω = 0.803. Given that the factor structure was obtained by exploratory factor analysis in the same sample in which the main analyses were conducted, the obtained dimensions should be interpreted with caution as exploratory and sample-specific indicators of interpersonal communication skills.

#### 2.3.3. Active Empathic Listening Scale

Empathic listening was assessed using an adapted version of the Active Empathic Listening Scale by Bodie [11]. The instrument consists of 11 items assessed on a Likert scale from 1 to 7, with higher scores indicating more pronounced empathic listening. After linguistic and cultural adaptation, its factor structure was examined using exploratory factor analysis.

The suitability of the data for factor analysis was assessed using the Kaiser–Meyer–Olkin measure and Bartlett’s test of sphericity. The factor structure was examined using principal component analysis with oblique oblimin rotation. The number of factors was determined based on eigenvalues, scree plot inspection, theoretical interpretability, and item factor loadings. The complete pattern matrix and factor correlation matrix are provided in the Supplementary Materials.

Although the original version of the instrument includes three dimensions, exploratory factor analysis in this study indicated a two-factor structure. The obtained dimensions were noticing emotional and nonverbal cues and processing and responding. The noticing emotional and nonverbal cues dimension refers to the recognition of emotional and nonverbal aspects of communication, while the processing and responding dimension reflects the interpretation of communication content and the provision of an appropriate response.

The reliability of the subscales was assessed using McDonald’s omega coefficient. Omega coefficient values were ω = 0.894 for the noticing emotional and nonverbal cues dimension and ω = 0.928 for the processing and responding dimension. The overall reliability of the instrument was ω = 0.928. Since the obtained structure differs from the original structure of the instrument, the results related to this scale were interpreted as exploratory indicators of empathic listening in this sample.

### 2.4. Statistical Analysis

Descriptive statistics were used to describe the sample and study variables. Categorical variables are presented as absolute and relative frequencies, while numerical variables are presented as means and standard deviations. Distributional characteristics were examined before conducting the main analyses.

Associations between communication knowledge, empathic listening, and interpersonal communication skills were examined using Pearson or Spearman correlation coefficients, as appropriate. Four multiple linear regression models were conducted, one for each dimension of interpersonal communication skills. In all models, the predictors were knowledge of communication theory and principles, knowledge of communication processes and skills, processing and responding, and noticing emotional and nonverbal cues.

Regression models were interpreted as exploratory models of association and explained variance, not as causal models. Regression assumptions were checked using residual diagnostics, variance inflation factors, and the Durbin–Watson statistic. The level of statistical significance was set at *p* < 0.05. Analyses were performed in R, version 4.3.1, and JASP, version 0.93.0.

## 3. Results

A total of 138 healthcare professionals involved in oncology care participated in the study. The majority of participants were women (75.4%), and physicians represented the largest professional group (34.8%). The mean age of participants was 43.10 ± 12.41 years, with an average of 20.49 ± 12.33 years of total professional experience and 14.95 ± 11.38 years of oncology care experience (Table 1).

Descriptive statistics for the study variables are presented in Table 2. Among the dimensions of interpersonal communication skills, clear message delivery and assertiveness showed the highest mean score, whereas emotional interaction management showed the lowest mean score. Regarding empathic listening, processing and responding demonstrated slightly higher scores than noticing emotional and nonverbal cues.

Correlation analysis revealed several statistically significant associations (Table 3). Knowledge of communication processes and skills showed a weak positive correlation with clear message delivery and assertiveness and a weak negative correlation with active listening. Both empathic listening dimensions were positively associated with clear message delivery and assertiveness. In contrast, processing and responding and noticing emotional and nonverbal cues were negatively associated with emotional interaction management. No significant associations were observed between knowledge of communication theories and principles and any communication skills dimension.

To examine the extent to which communication knowledge and empathic listening were associated with interpersonal communication skills, four multiple linear regression analyses were performed. All models included knowledge of communication theories and principles, knowledge of communication processes and skills, processing and responding, and noticing emotional and nonverbal cues as predictors. Regression analyses were conducted on 136 participants with complete data. The models were interpreted as exploratory models of association and explained variance rather than causal models.

The model predicting clear message delivery and assertiveness was statistically significant and explained 13.4% of the variance. Processing and responding was the only statistically significant predictor. The model predicting emotional interaction management was also statistically significant and explained 7.6% of the variance. Noticing emotional and nonverbal cues was the only statistically significant predictor and was negatively associated with emotional interaction management. The models predicting active listening, communication flow and feedback management were not statistically significant. The full regression results are presented in Table 4.

## 4. Discussion

This study examined the association between communication knowledge, empathic listening, and interpersonal communication skills among healthcare professionals involved in oncology care. The main findings show that selected dimensions of empathic listening were associated with selected dimensions of self-assessed communication skills, while communication knowledge, measured using a questionnaire developed for the purposes of this study, was not independently associated with dimensions of interpersonal communication skills. These findings should be interpreted with caution because the explained variance of the regression models was low, and the measures used were exploratory and self-reported in nature.

The most consistent finding was the positive association between processing and responding and clear message delivery and assertiveness. Participants who rated their ability to process patients’ verbal and emotional messages and provide an appropriate response more highly also rated their own clarity and assertiveness in communication more highly. This finding suggests that active processing of communication content may be associated with clearer message structuring and more confident expression in communication. In the oncology context, this may be relevant because healthcare professionals often need to provide clear information, respond to emotional reactions, and support patient participation in care [6,7,8].

However, this result should not be interpreted as evidence of a strong predictive effect of empathic listening. The model accounting for clear message delivery and assertiveness explained 13.4% of the variance, indicating that most of the variability in this communication skill remains unexplained by the included predictors. Therefore, the results can be viewed as indicating a limited but potentially relevant association between selected aspects of empathic listening and self-reported communication skills.

Communication knowledge was not a statistically significant independent predictor of any dimension of interpersonal communication skills. This finding does not mean that communication knowledge is not important for professional communication. A theoretical understanding of communication principles is an important basis for structured, professional, and ethically appropriate communication with patients [8,10]. However, the results of this study suggest that declarative knowledge, as operationalized in this study, was not sufficiently related to self-assessed practical communication skills. It is possible that the transfer of knowledge into clinical behavior requires experiential learning, practice, reflection, supervision, and feedback [18,19,20,21,22,23].

This finding should also be interpreted with regard to the characteristics of the communication knowledge questionnaire used. The questionnaire was developed for the purposes of this study and was not previously externally validated. Although it showed satisfactory internal consistency in this sample, its construct, criterion, and content validity should be further examined. Therefore, it is possible that the lack of association between communication knowledge and communication skills partly reflects the characteristics of the measurement instrument itself, and not necessarily a true lack of association between knowledge and communication practice.

The negative association between noticing emotional and nonverbal cues and emotional interaction management requires particularly cautious interpretation. This finding should not be interpreted as an indication that greater emotional perceptiveness impairs communication ability. One possible explanation is that healthcare professionals who are more attentive to emotional and nonverbal cues may be more aware of the complexity of emotional interactions with patients and therefore more critical when assessing their own ability to manage them. However, other explanations are also possible. The finding may reflect emotional burden associated with oncology care, differences in professional self-confidence, the specificity of self-assessment, or the psychometric properties of the exploratory emotional interaction management dimension. Since all data were collected through self-report, it is not possible to conclude whether this finding reflects actual communication behavior or the way in which participants assess their own competence [27].

In general, self-assessment of professional competencies may be influenced by subjective judgment, self-criticism, and the individual’s limited ability to accurately assess their own performance [27]. Therefore, future studies should include patient assessments, observer assessments, simulated clinical interviews, or analysis of real clinical communication situations to provide a more objective assessment of communication behavior.

The results support the importance of distinguishing between communication knowledge, empathic listening, and practical communication skills. In the education of healthcare professionals in oncology, it is not enough to focus only on the transfer of theoretical knowledge. Educational programs should include experiential methods, simulated conversations, structured feedback, reflective practice, and practicing responses to emotionally demanding situations, as these approaches are consistent with previous research on communication skills training and reflective practice in healthcare education [18,19,20,21,22,23]. However, given the exploratory nature of this study and the low explained variance of the models, the practical implications should be interpreted as directions for further research and education development rather than as firm evidence of the effectiveness of specific educational approaches.

It is also important to highlight the psychometric limitations of the instruments used. The adapted versions of the instruments showed factor structures that differed from the original versions, and the dimensions used in the analyses were obtained by exploratory factor analysis in the same sample in which the main analyses were conducted. In addition, the sampling adequacy for the adapted ICSI was acceptable but modest, which may have affected the stability of the extracted factor structure. Such an approach allowed the empirically based formation of dimensions appropriate for this sample, but at the same time increases the risk of factor structure instability and possible overfitting to the data. Therefore, the obtained dimensions should be viewed as exploratory and sample-specific. Future research should validate the obtained structures using confirmatory factor analysis in larger and independent samples [28].

Study limitations should be considered when interpreting the findings. First, the cross-sectional design prevents conclusions about causal relationships between communication knowledge, empathic listening, and communication skills. Second, all main variables were assessed using self-reported measures, which may be influenced by socially desirable responding, subjective self-assessment, different levels of self-criticism, and common method bias. Third, the use of a convenience online sample and the response rate of 39.4% limit the generalizability of the findings to all healthcare professionals involved in oncology care. It is possible that professionals with greater interest in communication or oncology communication training were more likely to participate. Fourth, the sample included different professional groups, including physicians, nurses, and other healthcare professionals, whose communication roles and responsibilities may differ. Finally, the adapted instruments were examined using exploratory factor analysis in the same sample in which the main correlation and regression analyses were conducted. This may increase the risk of sample-specific factor solutions and overfitting. Therefore, the findings should be interpreted as exploratory and should be confirmed in larger, independent samples using externally validated instruments and more objective indicators of communication behavior.

Despite these limitations, the study contributes to understanding the association between declarative communication knowledge, empathic listening, and self-reported communication skills in the specific context of oncology care. Its value is primarily exploratory. The results can serve as a starting point for further research, psychometric validation of adapted instruments, and the development of educational programs that link theoretical knowledge with experiential learning and reflective practice.

Future research should include larger and independent samples of healthcare professionals, confirm the factor structures of the instruments used, further validate the communication knowledge questionnaire, and examine the association of communication competencies with outcomes from the patient perspective. Longitudinal and interventional studies are needed to determine whether educational programs focused on empathic listening, simulations, and structured feedback can improve actual communication behavior in oncology care [18,19,20].

## 5. Conclusions

The results of this study showed that selected dimensions of empathic listening were associated with self-rated interpersonal communication skills among healthcare professionals involved in oncology care, whereas communication knowledge did not show an independent association with the observed dimensions of communication skills. The most pronounced association was found between processing and responding and clear message delivery and assertiveness, while noticing emotional and nonverbal cues was negatively associated with emotional interaction management.

Although these associations were statistically significant, the regression models explained only a relatively small proportion of the variance in communication skills. Therefore, the findings should be interpreted as exploratory indicators of associations between communication knowledge, empathic listening, and self-reported communication skills, rather than as evidence of causal relationships or strong predictive effects.

The findings suggest that relational aspects of communication, particularly empathic listening, may be relevant in the context of communication with oncology patients. However, this interpretation should be considered in light of the study limitations, including the cross-sectional design, reliance on self-reported measures, use of adapted and exploratory instruments, convenience sampling, and factor structures derived from the same study sample.

Further research using larger and independent samples, externally validated instruments, longitudinal designs, and more objective indicators of communication behavior is needed to confirm these associations and clarify their relevance for communication education in oncology care.

## Figures and Tables

**Table 1:** Sociodemographic and Professional Characteristics of Participants.

		*n* (%)
Gender	Men	33 (24.1)
	Women	104 (75.4)
Profession	Physician	47 (34.8)
	Master of Nursing	34 (25.2)
	Bachelor of Nursing	25 (18.5)
	Nurse/Medical Technician	27 (20.0)
	Auxiliary Healthcare Worker	1 (0.7)
Education Level	Higher Education	82 (61.2)
	Professional/Undergraduate Degree	25 (18.7)
	Secondary Education	27 (20.1)
		M ± SD
Age, years		43.10 ± 12.41
Total Work Experience, years		20.49 ± 12.33
Oncology Care Experience, years		14.95 ± 11.38

Note: *n* = number of participants; % = percentage; M = mean; SD = standard deviation.

**Table 2:** Descriptive Statistics of Study Variables.

	N	Minimum	Maximum	M ± SD
Knowledge of communication theories and principles	136	0.00	7.00	2.19 ± 2.05
Knowledge of communication processes and skills	136	0.00	12.00	8.67 ± 2.01
Clear message delivery and assertiveness	138	0.20	2.85	2.08 ± 0.49
Active listening	138	0.14	3.00	1.43 ± 0.64
Communication flow and feedback management	138	0.00	3.00	1.57 ± 0.76
Emotional interaction management	138	0.00	3.00	1.33 ± 0.87
Processing and responding	136	1.86	7.00	5.14 ± 0.98
Noticing emotional and nonverbal cues	136	1.00	7.00	4.94 ± 1.04

Note: N = number of participants; M = mean; SD = standard deviation.

**Table 3:** Correlations Between Communication Knowledge, Empathic Listening, and Interpersonal Communication Skills.

		Clear Message Delivery and Assertiveness	Active Listening	Communication Flow and Feedback Management	Emotional Interaction Management
Knowledge of communication theories and principles	ρ	−0.034	−0.075	−0.139	−0.044
	*p*	0.690	0.383	0.106	0.610
Knowledge of communication processes and skills	ρ	0.180	−0.179	−0.072	−0.139
	*p*	0.036	0.038	0.403	0.107
Processing and responding	ρ	0.349	−0.128	−0.104	−0.251
	*p*	<0.001	0.136	0.230	0.003
Noticing emotional and nonverbal cues	ρ	0.191	−0.082	−0.096	−0.218
	*p*	0.026	0.343	0.265	0.011

Note. ρ = Spearman correlation coefficient, as appropriate; *p* = statistical significance.

**Table 4:** Multiple Linear Regression Models Predicting Dimensions of Interpersonal Communication Skills.

	B	SE	95% CI for B	β	t	*p*	VIF
Clear message delivery and assertiveness							
Knowledge of communication theories and principles	−0.002	0.019	−0.041, 0.036	−0.009	−0.116	0.908	1.012
Knowledge of communication processes and skills	0.026	0.020	−0.014, 0.067	0.108	1.291	0.199	1.061
Processing and responding	0.180	0.052	0.077, 0.283	0.361	3.444	0.001	1.666
Noticing emotional and nonverbal cues	−0.027	0.049	−0.125, 0.070	−0.058	−0.556	0.579	1.661
Active listening							
Knowledge of communication theories and principles	−0.025	0.027	−0.079, 0.028	−0.081	−0.937	0.351	1.012
Knowledge of communication processes and skills	−0.048	0.028	−0.103, 0.008	−0.148	−1.680	0.095	1.061
Processing and responding	−0.073	0.073	−0.216, 0.071	−0.110	−1.001	0.319	1.666
Noticing emotional and nonverbal cues	0.006	0.068	−0.129, 0.142	0.010	0.094	0.926	1.661
Communication flow and feedback management							
Knowledge of communication theories and principles	−0.056	0.032	−0.120, 0.007	−0.150	−1.745	0.083	1.012
Knowledge of communication processes and skills	−0.017	0.034	−0.084, 0.049	−0.046	−0.518	0.605	1.061
Processing and responding	−0.057	0.086	−0.227, 0.113	−0.073	−0.662	0.509	1.666
Noticing emotional and nonverbal cues	−0.040	0.081	−0.201, 0.120	−0.055	−0.497	0.620	1.661
Emotional interaction management							
Knowledge of communication theories and principles	−0.027	0.036	−0.099, 0.044	−0.064	−0.757	0.450	1.012
Knowledge of communication processes and skills	−0.042	0.038	−0.117, 0.033	−0.096	−1.109	0.270	1.061
Processing and responding	−0.004	0.097	−0.196, 0.188	−0.005	−0.043	0.966	1.666
Noticing emotional and nonverbal cues	−0.198	0.091	−0.379, −0.017	−0.234	−2.166	0.032	1.661

Note: B = unstandardized regression coefficient; SE = standard error; CI = confidence interval; β = standardized regression coefficient; t = *t*-test value; *p* = statistical significance; VIF = variance inflation factor. Model summaries: clear message delivery and assertiveness: R^2^ = 0.134, adjusted R^2^ = 0.108, F(4, 131) = 5.077, *p* = 0.001; active listening: R^2^ = 0.045, adjusted R^2^ = 0.015, F(4, 131) = 1.526, *p* = 0.198; communication flow and feedback management: R^2^ = 0.037, adjusted R^2^ = 0.008, F(4, 131) = 1.270, *p* = 0.285; emotional interaction management: R^2^ = 0.076, adjusted R^2^ = 0.048, F(4, 131) = 2.713, *p* = 0.033.

## Data Availability

The data are not publicly available due to ethical and privacy restrictions. Although the dataset has been anonymized, it contains information collected from healthcare professionals working in a specific clinical field and setting, and public sharing could potentially compromise participant confidentiality. Therefore, the data are available from the corresponding authors upon reasonable request and with appropriate ethical considerations.

## Supplementary Materials

The following supporting information can be downloaded at https://www.mdpi.com/article/10.3390/healthcare14131842/s1, Detailed exploratory factor analysis results are provided in the Supplementary Materials, Table S1: Kaiser–Meyer–Olkin Measure and Bartlett’s Test of Sphericity for the Adapted Interpersonal Communication Skills Inventory; Table S2: Eigenvalues and Explained Variance of the Extracted Factors of the Adapted Interpersonal Communication Skills Inventory; Table S3: Pattern Matrix of the Adapted Interpersonal Communication Skills Inventory; Table S4: Component Correlation Matrix of the Adapted Interpersonal Communication Skills Inventory; Table S5: Internal Consistency Reliability of the Adapted Interpersonal Communication Skills Inventory; Table S6: Kaiser–Meyer–Olkin Measure and Bartlett’s Test of Sphericity for the Adapted Active Empathic Listening Scale; Table S7: Eigenvalues and Explained Variance of the Extracted Factors of the Adapted Active Empathic Listening Scale; Table S8: Pattern Matrix of the Adapted Active Empathic Listening Scale; Table S9: Component Correlation Matrix of the Adapted Active Empathic Listening Scale; Table S10: Internal Consistency Reliability of the Adapted Active Empathic Listening Scale; Table S11: Kaiser–Meyer–Olkin Measure and Bartlett’s Test of Sphericity for the Communication Knowledge Questionnaire; Table S12: Eigenvalues and Explained Variance of the Extracted Factors of the Communication Knowledge Questionnaire; Table S13: Pattern Matrix of the Communication Knowledge Questionnaire; Table S14: Component Correlation Matrix of the Communication Knowledge Questionnaire; Table S15: Internal Consistency Reliability of the Communication Knowledge Questionnaire.
