# Supplementary material for: Empathic Listening and Communication Competencies Among Oncology Healthcare Professionals in Croatia: A Cross-Sectional Study Conducted in 2025
Source: Healthcare (Basel). 2026 Jun 24;14(13):1842. doi: 10.3390/healthcare14131842 (PMC13361448; doi:10.3390/healthcare14131842)
Supplement: Supplementary file 1 [file healthcare-14-01842-s001.zip › healthcare-4383041-supplementary.pdf]

Supplementary Materials for

Empathic Listening and Communication Competencies among Healthcare Professionals Involved in Oncology Care in Croatia: A Cross-Sectional Study Conducted in 2025

Overview

These supplementary materials provide detailed psychometric results for the instruments used in the study. They include exploratory factor analyses, factor loadings, component correlation matrices, reliability coefficients, and information on retained and excluded items. The analyses were conducted to examine the empirical structure of the adapted instruments in a sample of healthcare professionals involved in oncology care in Croatia. The resulting dimensions were used in the main analyses reported in the manuscript and should be interpreted as exploratory and sample-specific constructs.

Supplementary Table S1. Kaiser–Meyer–Olkin Measure and Bartlett's Test of Sphericity for the Adapted Interpersonal Communication Skills Inventory

Indicator	ICSI-40	ICSI-33
Kaiser–Meyer–Olkin measure	0.596	0.615
Bartlett's test of sphericity, χ^2	1316.745	1022.481
df	780	528
p	<0.001	<0.001

Note. ICSI = Interpersonal Communication Skills Inventory; KMO = Kaiser–Meyer–Olkin measure of sampling adequacy; χ^2 = chi-square value for Bartlett's test of sphericity; df = degrees of freedom; p = statistical significance.

Supplementary Table S2. Eigenvalues and Explained Variance of the Extracted Factors of the Adapted Interpersonal Communication Skills Inventory

Factor	SS loadings (λ)	% variance	Cumulative % variance	% extracted variance	Cumulative % extracted variance
F1. Clear message delivery and assertiveness	2.215	6.71	6.71	28.45	28.45
F2. Active listening	2.061	6.25	12.96	26.47	54.92
F3. Communication flow and feedback management	1.765	5.35	18.31	22.66	77.59
F4. Emotional interaction management	1.745	5.29	23.59	22.41	100.00

Note. λ = eigenvalue; % variance = percentage of total variance explained by each factor; cumulative % variance = cumulative percentage of total explained variance; % extracted variance = percentage of variance explained after extraction.

Supplementary Table S3. Pattern Matrix of the Adapted Interpersonal Communication Skills Inventory

Item	Item wording	F1	F2	F3	F4	Assigned factor
1	Difficulty talking with other people	0.120	0.150	0.080	0.210	—
2	Others put words in my mouth	0.280	-0.020	-0.450	0.692	F4
3	My words sound the way I want them to	0.472	0.078	0.045	-0.333	F1
4	Difficulty expressing ideas when they differ from others' ideas	-0.020	0.045	-0.480	0.579	F4
5	I assume that the other person knows what I mean	0.011	0.065	-0.480	0.517	F4
6	Others are interested while I am speaking	0.415	0.122	0.088	-0.381	F1
7	I easily recognize how others react	0.520	-0.033	0.120	-0.353	F1
8	I ask the other person for their opinion about the point I am trying to make	0.374	-0.110	0.040	0.033	F1
9	I am aware of how my tone of voice affects others	0.210	0.044	0.110	0.055	—
10	I try to talk about shared interests	0.549	0.023	0.111	-0.045	F1
11	I tend to talk more than the other person	0.070	0.122	0.506	0.070	F3
12	I ask questions when I do not understand	0.414	-0.005	0.120	0.044	F1
13	I try to figure out what the other person is going to say	-0.060	-0.022	0.648	-0.035	F3
14	I do not pay attention while talking	0.050	0.703	-0.005	0.045	F2
15	Difference between what a person says and how they feel	0.180	0.090	0.060	0.110	—
16	I clarify what I heard before offering a response	0.449	-0.050	0.155	0.022	F1
17	I finish sentences instead of other people	0.080	0.545	0.327	0.077	F2
18	I focus on facts and miss the emotional tone	0.045	0.220	0.110	0.088	—
19	I allow the other person to finish their thought	-0.050	0.306	-0.290	0.311	F4
20	Difficulty seeing things from another person's perspective	0.065	-0.060	0.331	0.050	F3
21	Difficulty hearing or accepting constructive criticism	0.150	0.188	0.090	0.030	—
22	I refrain from saying something that might upset others	0.088	0.110	0.250	0.140	—
23	When someone hurts me, I talk about it	0.317	0.155	-0.030	-0.075	F1
24	I try to put myself in the other person's situation	0.324	-0.088	0.080	-0.020	F1
25	I feel uncomfortable when I receive a compliment	-0.055	-0.033	0.447	0.030	F3
26	Difficulty disagreeing because of fear of anger	0.030	0.280	0.045	0.090	—
27	I find it difficult to give a compliment or praise	-0.030	0.788	-0.030	0.011	F2
28	Others notice that I think I am right	-0.045	0.348	0.110	0.210	F2
29	Others become defensive when I disagree	0.304	0.426	-0.090	0.111	F2
30	I help others understand me by expressing	0.511	-0.044	-0.060	0.112	F1

	how I feel					
31	I tend to change the subject when feelings enter the discussion	0.080	-0.020	0.612	0.040	F3
32	Difficulty thinking clearly when I am angry	0.022	0.427	0.150	0.020	F2
33	I become very upset when someone disagrees with me	0.170	0.170	-0.370	0.564	F4
34	Discussing a problem without anger	0.700	0.112	-0.045	0.088	F1
35	Satisfaction with how I respond to differences	0.476	-0.120	0.090	0.055	F1
36	I stay angry for a long time when someone upsets me	-0.075	0.346	0.376	0.220	F3
37	I apologize to a person whose feelings I have hurt	0.250	-0.386	-0.066	-0.340	F2
38	I admit when I am wrong	0.555	-0.012	0.140	0.098	F1
39	I avoid the topic if someone expresses feelings	-0.150	0.200	0.392	0.188	F3
40	Difficulty continuing a conversation when someone becomes upset	-0.090	0.055	0.457	0.300	F3

Note. ICSI = Interpersonal Communication Skills Inventory; F1 = clear message delivery and assertiveness; F2 = active listening; F3 = communication flow and feedback management; F4 = emotional interaction management. Items 1, 9, 15, 18, 21, 22, and 26 were excluded from the final ICSI-33 because they did not meet the factor retention criteria.

Supplementary Table S4. Component Correlation Matrix of the Adapted Interpersonal Communication Skills Inventory

Factor	F1	F2	F3	F4
F1. Clear message delivery and assertiveness	1.000			
F2. Active listening	0.147	1.000		
F3. Communication flow and feedback management	-0.176	-0.115	1.000	
F4. Emotional interaction management	-0.185	0.015	0.170	1.000

Note. F1–F4 = extracted components of the adapted Interpersonal Communication Skills Inventory.

Supplementary Table S5. Internal Consistency Reliability of the Adapted Interpersonal Communication Skills Inventory

Dimension	Number of items	McDonald's ω
F1. Clear message delivery and assertiveness	13	0.720
F2. Active listening	7	0.611
F3. Communication flow and feedback management	8	0.604
F4. Emotional interaction management	5	0.653
Total ICSI-33	33	0.803

Note. ICSI = Interpersonal Communication Skills Inventory; ω = McDonald's omega coefficient.

Supplementary Table S6. Kaiser–Meyer–Olkin Measure and Bartlett's Test of Sphericity for the Adapted Active Empathic Listening Scale

Indicator	Value
Kaiser–Meyer–Olkin measure	0.894
Bartlett's test of sphericity, χ^2	1039.314
df	55
p	<0.001

Note. KMO = Kaiser–Meyer–Olkin measure of sampling adequacy; χ^2 = chi-square value for Bartlett's test of sphericity; df = degrees of freedom; p = statistical significance.

Supplementary Table S7. Eigenvalues and Explained Variance of the Extracted Factors of the Adapted Active Empathic Listening Scale

Factor	Eigenvalue (λ)	% variance	Cumulative % variance	Extraction sum of squared loadings
F1. Processing and responding	6.398	58.160	58.160	5.862
F2. Noticing emotional and nonverbal cues	1.404	12.765	70.925	4.599
Total	7.802	70.925	—	10.461

Note. AELS = Active Empathic Listening Scale; λ = eigenvalue; % variance = percentage of total variance explained by each factor.

Supplementary Table S8. Pattern Matrix of the Adapted Active Empathic Listening Scale

Item wording	F1	F2
Responding: I let others know that I will remember what they said.	0.919	-0.139
Responding: I show others through body language that I am listening, such as nodding.	0.911	-0.072
Responding: I let others know that I am open to their ideas.	0.863	0.018
Responding: I ask questions that show I understand the situation.	0.850	0.022
Processing: I take others' viewpoints into account.	0.784	0.088
Processing: I let others know that I will remember what they said.	0.708	0.133
Processing: I summarize points of agreement and disagreement.	0.564	0.228
Noticing: I am aware of what others imply but do not say.	-0.072	0.909
Noticing: I can sense what others do not say.	-0.065	0.898
Noticing: I understand how others feel.	0.162	0.761
Noticing: I listen for more than spoken words.	0.217	0.714

Note. AELS = Active Empathic Listening Scale; F1 = processing and responding; F2 = noticing emotional and nonverbal cues. Values represent factor loadings after oblimin rotation.

Supplementary Table S9. Component Correlation Matrix of the Adapted Active Empathic Listening Scale

Factor	F1	F2
F1. Processing and responding	1.000	0.559
F2. Noticing emotional and nonverbal cues	0.559	1.000

Note. AELS = Active Empathic Listening Scale; F1 = processing and responding; F2 = noticing emotional and nonverbal cues.

Supplementary Table S10. Internal Consistency Reliability of the Adapted Active Empathic Listening Scale

Dimension / scale	Number of items	McDonald's ω
Processing and responding	7	0.928
Noticing emotional and nonverbal cues	4	0.894
Total AELS	11	0.926

Note. AELS = Active Empathic Listening Scale; α = Cronbach's alpha coefficient.

Supplementary Table S11. Kaiser–Meyer–Olkin Measure and Bartlett's Test of Sphericity for the Communication Knowledge Questionnaire

Indicator	Value
Kaiser–Meyer–Olkin measure	0.795
Bartlett's test of sphericity, χ^2	1328.946
df	190
p	<0.001

Note. KMO = Kaiser–Meyer–Olkin measure of sampling adequacy; χ^2 = chi-square value for Bartlett's test of sphericity; df = degrees of freedom; p = statistical significance.

Supplementary Table S12. Eigenvalues and Explained Variance of the Extracted Factors of the Communication Knowledge Questionnaire

Factor	SS loadings (λ)	% variance	Cumulative % variance	% extracted variance	Cumulative % extracted variance
F1. Knowledge of communication theory and principles	6.334	31.67	31.67	29.88	29.88
F2. Knowledge of communication processes and skills	4.947	24.73	56.40	22.06	51.94

Note. λ = eigenvalue; % variance = percentage of total variance explained by each factor; cumulative % variance = cumulative percentage of total explained variance; % extracted variance = percentage of variance explained after extraction.

Supplementary Table S13. Pattern Matrix of the Communication Knowledge Questionnaire

Item wording	F1	F2
Is there such a thing as excessive communication, such as providing too much information in a conversation?	-0.750	0.264
In conversation, it is assumed that patients know the basics and facts.	0.971	0.045
In a team, it is assumed that all members know all information and tasks.	0.948	-0.132
The message shared by a physician or nurse should be adapted to the patient.	0.156	0.752
Professionals with good skills will rarely experience misunderstanding.	-0.031	0.306
Professionals with good skills will never experience misunderstanding.	0.061	0.290
We can speak in two ways and listen in ten ways.	-0.081	0.344
Selective listening leads to misunderstandings in communication.	-0.216	0.513
Active listening is poor because it means interrupting the person who is	0.939	0.176

speaking.		
Passive listening is a desirable way of listening because it helps us remember better.	0.849	0.034
Good communication involves asking questions when something is unclear.	-0.249	0.675
If a patient does not understand the message or understands it incorrectly, it is the patient's fault.	0.892	0.075
The main task in communication is to achieve understanding.	-0.138	0.795
Speaking from one's own position usually disrupts the natural flow of communication.	0.028	0.271
It is good to mention the patient's name during communication.	-0.090	0.788
Every message we send in a conversation has only one side.	0.967	0.139
It is good to be assertive and stand up for oneself in communication.	-0.018	0.537
In disagreement, it is best to withdraw from the discussion as soon as possible.	0.099	0.323
A professional should always follow their prepared technique when delivering information.	0.062	0.212
Communication skills are necessary for high-quality oncology care.	0.229	0.960

Note. F1 = knowledge of communication theory and principles; F2 = knowledge of communication processes and skills. Values represent factor loadings based on exploratory factor analysis using a tetrachoric correlation matrix. Items with insufficient loadings were excluded from the final score.

Supplementary Table S14. Component Correlation Matrix of the Communication Knowledge Questionnaire

Factor pair	Correlation
F1. Knowledge of communication theory and principles with F2. Knowledge of communication processes and skills	-0.041

Note. F1 = knowledge of communication theory and principles; F2 = knowledge of communication processes and skills.

Supplementary Table S15. Internal Consistency Reliability of the Communication Knowledge Questionnaire

Dimension / questionnaire	Number of items	McDonald's ω
Knowledge of communication theory and principles	7	0.981
Knowledge of communication processes and skills	10	0.876
Total Communication Knowledge Questionnaire	17	0.909

Note. ω = McDonald's omega coefficient.
